# Systematic collection, annotation, and pattern analysis of viral vaccines in the VIOLIN vaccine knowledgebase

**DOI:** 10.3389/fcimb.2025.1509226

**Published:** 2025-02-07

**Authors:** Anthony Huffman, Mehul Gautam, Arya Gandhi, Priscilla Du, Lauren Austin, Kallan Roan, Jie Zheng, Yongqun He

**Affiliations:** ^1^ Department of Computational Medicine and Bioinformatics, University of Michigan Medical School, Ann Arbor, MI, United States; ^2^ College of Literature, Science, and the Arts, University of Michigan, Ann Arbor, MI, United States; ^3^ Unit for Laboratory Animal Medicine, University of Michigan, Ann Arbor, MI, United States

**Keywords:** virus, ontology, gene enrichment, reverse vaccinology, antigens, adhesin, VIOLIN vaccine knowledgebase, Vaxign-ML

## Abstract

**Background:**

Viral vaccines have been proven significant in protecting us against viral diseases such as COVID-19. To better understand and design viral vaccines, it is critical to systematically collect, annotate, and analyse various viral vaccines and identify enriched patterns from these viral vaccines.

**Methods:**

We systematically collected experimentally verified viral vaccines from the literature, manually annotated, and stored the information in the VIOLIN vaccine database. The annotated information included basic vaccine names, pathogens and diseases, vaccine components, vaccine formulations, and their induced host responses. Enriched patterns were identified from our systematical analysis of the viral vaccines and vaccine antigens.

**Results:**

A total of 2,847 viral vaccines against 95 viral species (including 72 RNA viral species and 23 DNA viral species) were collected, manually annotated, and stored in the VIOLIN vaccine database. These viral vaccines used 542 vaccine antigens. A taxonomical analysis found various DNA and RNA viruses covered by the viral vaccines. These vaccines target different viral life cycle stages (e.g., viral entry, assembly, exit, and immune evasion) as identified in top ranked human, animal vaccines, and HPV vaccines. The vaccine antigen proteins also show up in different virion locations in viruses such as HRSV vaccines. Both structural and non-structural viral proteins have been used for viral vaccine development. Protective vaccine antigens tend to have a protegenicity score of >85% based on the Vaxign-ML calculation, which measures predicted suitability for vaccine use. While predicted adhesins still have significantly higher chances of being protective antigens, only 21.42% of protective viral vaccine antigens were predicted to be adhesins. Furthermore, our Gene Ontology (GO) enrichment analysis using a customized Fisher’s exact test identified many enriched patterns such as viral entry into the host cell, DNA/RNA/ATP/ion binding, and suppression of host type 1 interferon-mediated signaling pathway. The viral vaccines and their associated entities and relations are ontologically modeled and represented in the Vaccine Ontology (VO). A VIOLIN web interface was developed to support user friendly queries of viral vaccines.

**Discussion:**

Viral vaccines were systematically collected and annotated in the VIOLIN vaccine knowledgebase, and the analysis of these viral vaccines identified many insightful patterns.

## 1 Introduction

Viral pathogens have posted dramatic threat to the public health. For example, the 1918 influenza pandemic killed 50 million or more people; the HIV/AIDS pandemic, initially recognized in 1981, has killed more than 37 million people (Morens and Fauci, 2020); and the recent COVID-19 viral pandemic has caused over 7 million deaths according to the WHO records as of December 2024 (https://data.who.int/dashboards/covid19/deaths?n=o). Viral pathogens may contain either DNA or RNA as their genome and they can infect all types of life forms including humans, animals, plants, and microorganisms. The virulence of a virus, i.e., the severity of the disease the pathogen can cause, can vary within a species (Balloux and Van Dorp, 2017). Viruses require a viable host in order for the viral pathogen to survive to use the host organism’s biological resources to replicate before exiting (Xiang et al., 2008).

A vaccine is a cost-effective and powerful immunization tool used to reduce the incidence of infectious diseases by simulating an effective immune response. Vaccines work by introducing antigens (e.g., such as protein antigens) to the host immune system. Different vaccine types exist. For example, subunit vaccines directly use pathogen subunit(s) such as proteins or peptides as vaccine antigen. Inactivated vaccines use chemically or heat-inactivated versions of the disease as the antigen. Live-attenuated vaccines use naturally or genetically mutated (attenuated) versions of the disease. Toxoid vaccines use an inactivated toxin (toxoid) produced from the disease as the antigen. Conjugate vaccines use proteins derived from the outer coat of the disease. DNA vaccines utilize the replicating DNA plasmids containing genetic material from the disease-causing pathogens. Recombinant vector vaccines use modified versions of different pathogens to encode the genes for microbial antigens presented to the host (Xiang et al., 2008). Lastly, mRNA vaccines use the messenger RNA (RNA) of a protective protein antigen(s) to stimulate the production of protective immunity against the antigen(s). These different methods of creating viral vaccines give humanity the power to fight the infectious disease.

To document and standardize vaccine related information, we have developed the web-based VIOLIN vaccine database, a comprehensive catalog of vaccines that are experimentally verified, in clinical trials, or have been approved for market use (Xiang et al., 2008; He et al., 2014). The VIOLIN database annotates vaccines with information on vaccine components, antigens, vaccine efficacy, vaccine safety, host response, and gene engineering. The manually annotated vaccines in VIOLIN include those licensed human and animal vaccines, vaccine candidates at the clinical stage, and vaccine candidates that were at least experimentally verified using laboratory animal medicine. Compiling the vaccines into a centralized database allows for the efficient retrieval of the vaccines through various VIOLIN search programs and provides researchers with data to facilitate the understanding of vaccines to fight infectious diseases (Xiang et al., 2008; He et al., 2014). The community-based Vaccine Ontology (VO) (Ozgur et al., 2011; Lin and He, 2012; He et al., 2014) was also developed and used to ontologically represent these vaccines, vaccine components, and their relations. VIOLIN also has a user-friendly web and data submission system that anyone with an account can use to submit vaccine data.

The VIOLIN vaccine resource has been used in many applications such as the leverage for development of safe efficacious vaccines (Khan et al., 2022) or understanding host immune response to vaccines (Berke et al., 2021). VIOLIN includes Protegen (Yang et al., 2011), a protective antigen database, which collects and manually annotated >1,600 protective antigens as of December 2024. Here a protective antigen is defined as an antigen experimentally verified to be capable of inducing protective adaptive immunity against a specific pathogen or the cause of a specific disease such as cancer (Yang et al., 2011). The Protegen database (Yang et al., 2011) data has been widely used as the gold standard of protective antigens for vaccine antigen prediction. The Protegen data has also been used to identify features within bacterial antigens as good predictors of protective vaccines (Ong et al., 2017a, 2020a). For example, one of the favorable predictors is the likelihood the protein can function as an adhesin, i.e. a class of proteins that can bind and interact with the cellular membrane of host cells (Gomez et al., 2013; Ong et al., 2017a; Sayers et al., 2019; Ong et al., 2020b).

This paper reports our systematic collection, annotation, and pattern analysis of viral vaccines and protective viral antigens using the VIOLIN bioinformatics pipeline. Using different bioinformatics methods, enriched patterns among all the hundreds of protective viral vaccine antigens were identified. We have also used the VO to ontologically represent these vaccines and developed web query interfaces for user-friendly queries.

## 2 Methods

**Figure 1:**
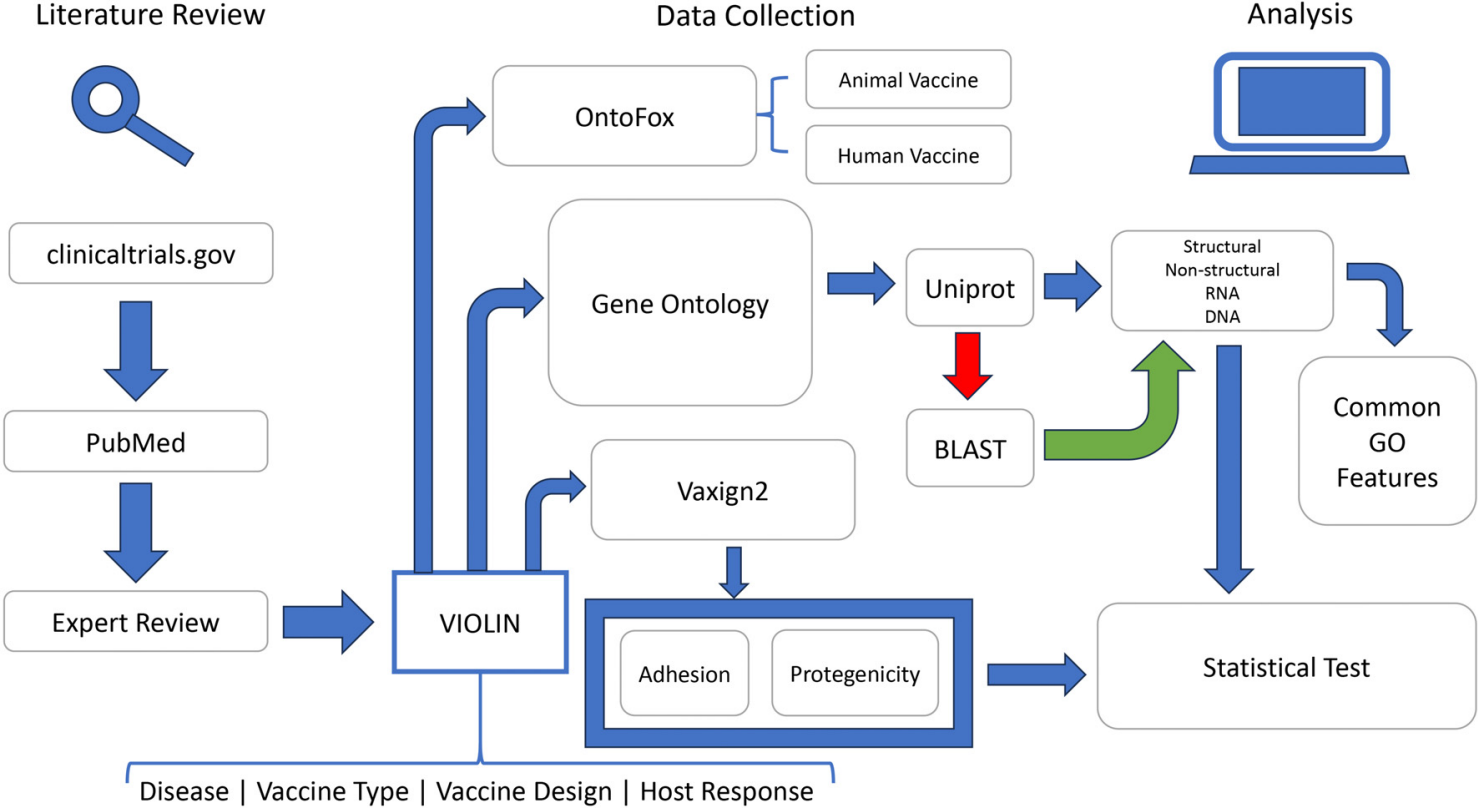
Overall study workflow. This flowchart depicted the entire process to train and evaluate machine learning-based reverse vaccinology models. See main text for details.

The overall project workflow is described in a figure below (**Figure 1**). Briefly, public data resources including PubMed and Clinicaltrials.org were annotated to extract the information of viral vaccines into the VIOLIN vaccine database. All the viral vaccine antigens were collected for different analyses. The Gene Ontology (GO) enrichment analysis was performed using the DAVID tool (Sherman et al., 2022) and our own Fisher’s extract test. The Vaxign2 vaccine design tool was used to calculate the adhesin probability and protegenicity score (Ong et al., 2021). The Vaccine Ontology (VO) semantically represents the information of all the vaccines including viral vaccines. The tool OntoFox (Xiang et al., 2010) was used to extract the information of only viral vaccines from the VO for further analysis.

### 2.1 Viral vaccine curation, storage, and representation

#### 2.1.1 Collection and annotation of viral vaccines and vaccine antigens into VIOLIN

New vaccines were annotated and recorded on VIOLIN using sources queried from PubMed or clinicaltrials.gov. When entering a vaccine into the VIOLIN database, PubMed or clinicaltrials.gov was often first used to find potential vaccines and simultaneously compared to the vaccines already entered in VIOLIN. When a new vaccine was found, PubMed was utilized to find related articles about the development and trials of the vaccine, and the appropriate information was input into the appropriate sections. The VIOLIN database includes which vaccine antigen was used in the construction of a viral vaccine. Each protein antigen was annotated with a corresponding NCBI gene ID and its associated protein ID. After submission, data is subject to review from domain experts and all data in the database is backed up daily.

#### 2.1.2 Proofreading and curation of viral vaccines in VIOLIN

After viral vaccines were submitted, an experienced domain expert proofread the annotated results. Only after the proofreading and approval, the submitted viral vaccine records could be queried and visualized by public users in VIOLIN.

#### 2.1.3 Ontological representation of viral vaccines

The Vaccine Ontology (VO) (Ozgur et al., 2011; Lin and He, 2012; He et al., 2014) was used to represent all the viral vaccines. All the viral vaccines in VIOLIN were represented, and VO IDs were assigned. In addition to viral vaccine labels and VO IDs, the VO also provides definitions, VIOLIN IDs, references, and many logical axioms for providing vaccine attributes such as vaccine components, qualities, and roles. Protege OWL editor (Musen and Protege, 2015) was used for manual VO editing and visualization. OntoFox (Xiang et al., 2010) and Ontorat (Xiang et al., 2015) were used for existing ontology term extraction for reuse and new term generation, respectively.

### 2.2 Viral vaccine and antigen data analysis

#### 2.2.1 Taxonomic analysis of viral vaccines

OntoFox (Xiang et al., 2010) was used to extract taxonomic organism terms from the NCBITaxon taxonomy ontology (Liu et al., 2017; Schoch et al., 2020). The ancestor terms of species and the hierarchical relations among different levels of taxonomic terms were also extracted using OntoFox using the setting “includeComputedIntermediates” (Xiang et al., 2010). Protege OWL editor was used for visualization. In addition, viruses were utilized in NCBITaxon to identify if they are DNA or RNA viruses. Finally, each viral protein identified as protective viral antigen was mapped to a UniProt ID for later analysis.

#### 2.2.2 Gene ontology term enrichment analysis

We developed and applied a Fisher’s exact test for the GO enrichment analysis. Specifically, GO features were extracted from UniProt ID using the listed protein identifier. GO cellular component proteins were utilized to identify proteins as structural or non-structural. For proteins without a GO cellular component, NCBI BLAST (Johnson et al., 2008) was utilized to find a protein with relevant ontology annotation. Finally, extraction of GO biological process and GO molecular functions were retrieved for later gene set analysis between structural and non-structural proteins using the UniProt KB API (Uniprot, 2023). Duplicates of the same protein were included if an ID mapped to multiple UniProt proteins. The GO annotations related to biological processes and molecular functions for these proteins were retrieved. A Python program was developed to extract and calculate the occurrence of each GO term. We developed a script to execute a Fisher’s exact test to determine if there were any statistically significant differences for the top 10 most common features found in structural and non-structural proteins. As UniProtKB did not consistently 1:1 map to proteins and lacked information on some proteins, the raw count of proteins used with the GO annotation differs from the number of antigens collected.

We classified protective viral antigens as structural or non-structural proteins based on GO cell component annotation. In addition, using GO biological process annotations, the temporal roles of these proteins were classified into the following categories: viral entry into host cell, viral assembly of capsid, viral exit of the virus, immune evasion, and unknown.

#### 2.2.3 In silico analysis of vaccine antigens using Vaxign2 and Vaxign-ML

The vaccine design tool Vaxign2 (Ong et al., 2021) and Vaxign-ML (Ong et al., 2020a) was used to identify similarities or useful predictive features among viral proteins. Two sets of viral proteins were used. The first set is the protective viral antigens that were collected as the antigen component of the viral vaccines. These protective viral antigens were also stored in the Protegen database (Yang et al., 2011). The second set is the collection of non-protective viral proteins collected from UniProt, based on the criteria of low protein sequence similarity (<30%) and no homology (BLASTp E-value ≤ 10E−3) to known protective viral proteins as defined in previous studies (Ong et al., 2020a, 2020b). Using the gene engineering information for the collected vaccines, the genetic sequences for different antigen proteins were pasted into the Vaxign2 dynamic analysis. Data was collected on adhesin probability and protegenicity score and documented for each of the antigen proteins. A 2-tailed t-test was used to determine if the distribution of adhesin probability and protegenicity between structural and non-structural proteins for RNA and DNA was statistically significant.

#### 2.2.4 Website query and analysis

The VIOLIN website (https://violinet.org) was used for web query analysis and tutorial on how to query for specific viral proteins.

## 3 Results

### 3.1 Collection, and analysis of viral vaccines in VIOLIN

**Figure 2:**
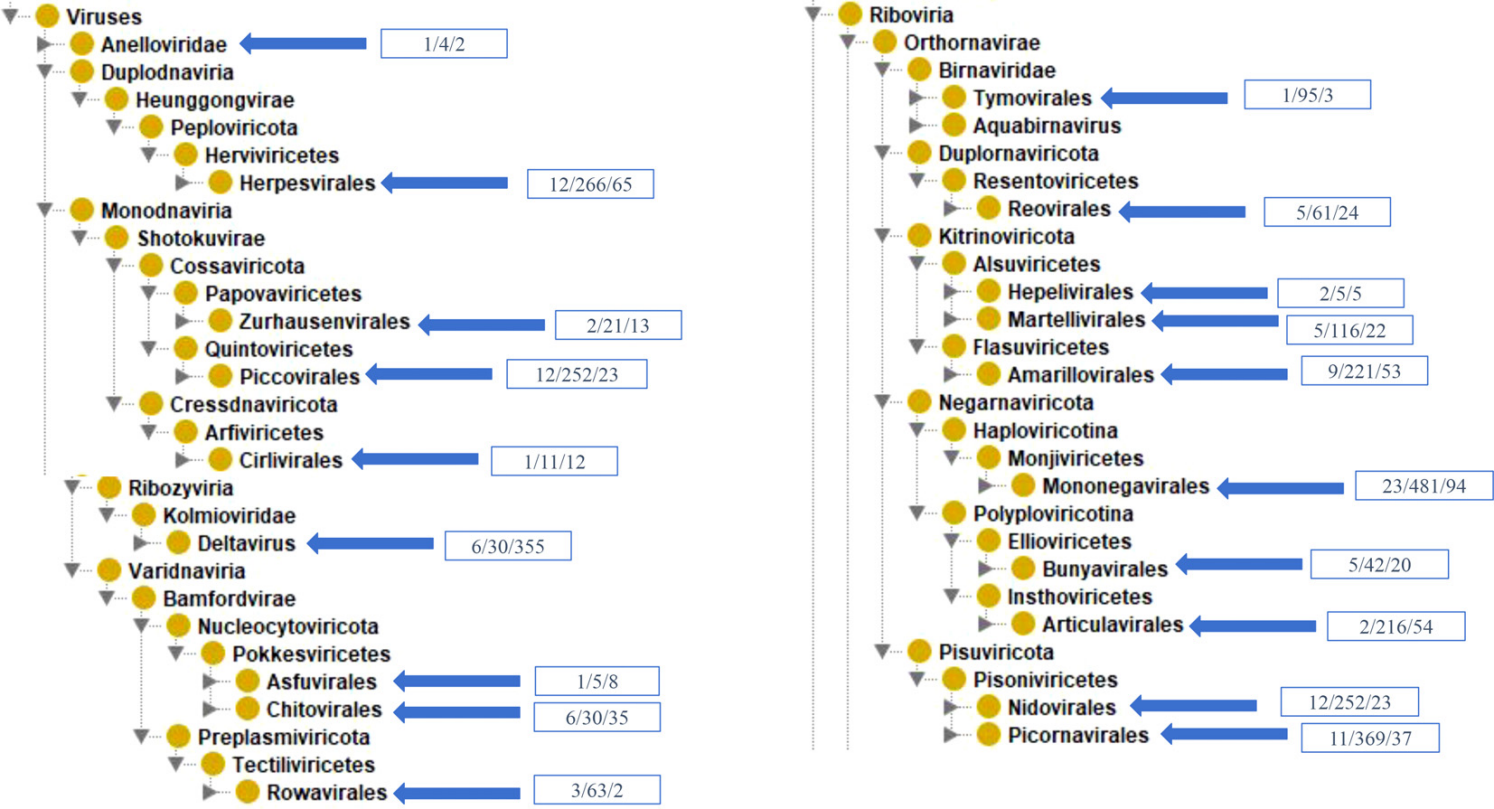
Taxonomic representation of vaccines in VIOLIN. Each box in blue contains the number of species, vaccines, and viral antigens, respectively.

VIOLIN contains 2,847 vaccines from 95 different viral species. These 95 species are part of 14 distinct viral clades collected in VIOLIN (**Supplementary Table S1**, **Figure 2**). Our taxonomical analysis found the vaccines were developed against both DNA and RNA viruses, where the DNA viruses have double-stranded DNA (dsDNA) viruses including *Duplodnaviria* and *Varidnaviria*, and single-stranded DNA (dsDNA) viruses in the realm *Monodnaviria*, and RNA viruses are in the realms of *Ribozyviria* and *Riboviria* (**Figure 2**). The majority (72, 76%) of new viral viruses in VIOLIN are *Riboviria* RNA viruses. The best represented order is *Mononegavirales* under *Riboviria* (**Figure 2**), which includes Ebola, measles, mumps, and rabies (Amarasinghe et al., 2019). Most of these vaccines tend to be live attenuated vaccines (10, ~37%). The remainder of the viral species were DNA viruses.

Among 95 viral species annotated in VIOLIN, 82 of them can infect a variety of animals including livestock (cattle, poultry) and domesticated animals (dogs), and 46 of them can infect humans (**Supplementary Table S1**). Among 2,847 vaccines collected, 1,190 vaccines target the 46 human-infecting viral species.

The top 10 viral pathogens with the highest number of human vaccines or animal vaccines are shown in **Tables 1** or **2**, respectively. The top 4 human viral pathogens include influenza virus (213 vaccines), SARS-CoV-2 (159 vaccines), infectious bronchitis virus (IBV) (89 vaccines), and Rabies virus (42 vaccines) (**Table 1**). The top 4 ranked animal viral pathogens include bovine herpesvirus 1 (159 vaccines), bovine viral diarrhea virus 1 (129 vaccines), bovine parainfluenza 3 virus (BPIV-3) (108 vaccines), and Newcastle disease virus (100 vaccines) (**Table 2**).

**Table 1:** Top 10 human pathogens with the highest number of vaccines collected in VIOLIN.

Viral pathogen (human disease)	No. of vaccines (licensed)	No. of pathogen genes
Influenza virus (influenza)	213	53
SARS-CoV-2 (COVID-19)	159	2
Infectious Bronchitis Virus (IBV Respiratory Disease)	89	6
Rabies virus (Rabies)	42	7
Human Immunodeficiency Virus (AIDS)	39	52
SARS-CoV (SARS)	37	5
Ebola virus (Ebola fever)	32	21
Herpes simplex virus type 1 and 2 (Herpes)	29	18
West Nile Virus (West Nile fever)	25	3
Marburg Virus (Hemorrhagic Fever)	24	23

**Table 2:** Top 10 animal pathogens with the highest number of vaccines collected in VIOLIN.

Viral pathogen (Animal disease)	No. of vaccines (licensed)	No. of pathogen genes
Bovine herpesvirus 1 (Infectious bovine rhinotracheitis)	159	9
Bovine viral diarrhea virus 1 (Bovine viral Diarrhea)	129	1
Bovine Parainfluenza 3 Virus (BPIV-3)	108	0
Newcastle disease virus (Newcastle disease)	100	3
Infectious Bursal Disease Virus (Infectious Bursal Disease)	93	3
Canine distemper virus (Canine distemper)	79	2
Canine parvovirus (Canine parvovirus infection)	68	2
Canine Adenovirus Type 2	57	0
Canine parainfluenza virus	56	0
Bovine Respiratory Syncytial Virus	55	6

The largest set of human vaccines exist for both endemic viruses and zoonotic viruses that have jumped from a non-human host to a human host. The most prevalent endemic viruses include influenza viruses and IBV (Bouvier and Palese, 2008). The zoonotic jump of new viruses can lead to rapid development of new vaccines, with the most dramatic case being the hundreds of SARS-CoV-2 vaccines being used and developed. Some vaccines target multiple viruses simultaneously, such as the MMR vaccine targeting the measles, mumps, and rubella virus (Shah et al., 2024).

Animal vaccines exist for livestock, pets, and model organisms. Most animal vaccines target livestock, including cattle (bovine herpesvirus, bovine viral diarrhea virus, bovine respiratory syncytial virus), poultry (Newcastle disease virus, fowlpox virus), horses (equine rotavirus), and pigs (African Swine Fever and porcine rotavirus). For pets, the number is smaller, with 12 viruses targeting dogs (e.g., canine distemper virus, canine parainfluenza virus) and cats (e.g., feline immunodeficiency virus, feline infectious peritonitis virus).

### 3.2 Viral vaccines targeting different stages of life cycles

**Figure 3:**
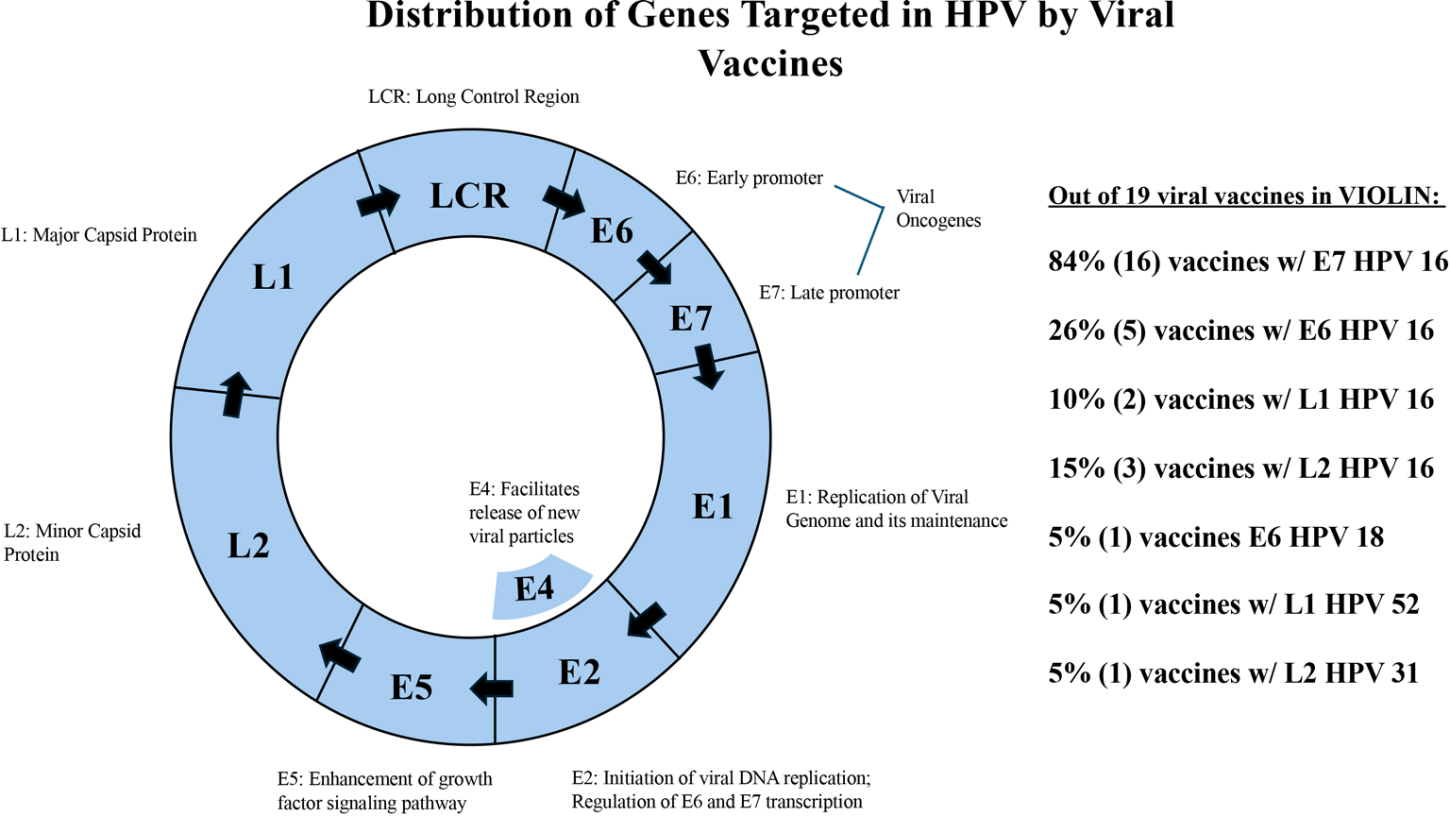
HPV vaccines targeting different stages of the viral life cycle. HPV is a single loop of RNA composed of capsid membrane proteins (L) and nonstructural proteins.

Our research found that viral vaccines often target specific stages in the viral replication cycles. Viral genomes tend to be small, and each protein has a clear role for its function. For example, the Human papillomavirus (HPV) is capable of inducing cervical and oropharyngeal cancers within humans (Graham, 2017). The most common HPV variants responsible for this are HPV 16 and HPV 18. There are 19 HPV vaccines collected in VIOLIN. **Figure 3** shows the genomic organizations of HPV. The majority of the HPV vaccines target E7 HPV 16 (84%) and E6 HPV 16 (26%) genes. HPV 16 E6/E7 are both vital for viral replication (Roman and Munger, 2013) and carcinogenic transformation (Peng et al., 2021). These proteins inhibit tumor suppressor p53 (Au Yeung et al., 2010). The remainder of HPV vaccines utilize L1 and L2 capsid proteins. L1 (Hernandez et al., 2011) is important in mediating cell attachment during infectious entry while L2 involves the virus entry into the cells and localization of viral components to the nucleus (Pereira et al., 2009). As such, vaccines that target the E proteins attempt to interfere with the antigen after infection, especially when the E proteins are expressed by carcinomic cells. The L protein vaccines, in contrast, attempt to target the virus during cell entry and replication of non-structural E proteins. By preventing localization of viral components to the nucleus, HPV does not replicate in the nucleus, therefore is a useful vaccine design. E1, E2, E4, E5 have not been targeted in any vaccine for HPV.

**Figure 4:**
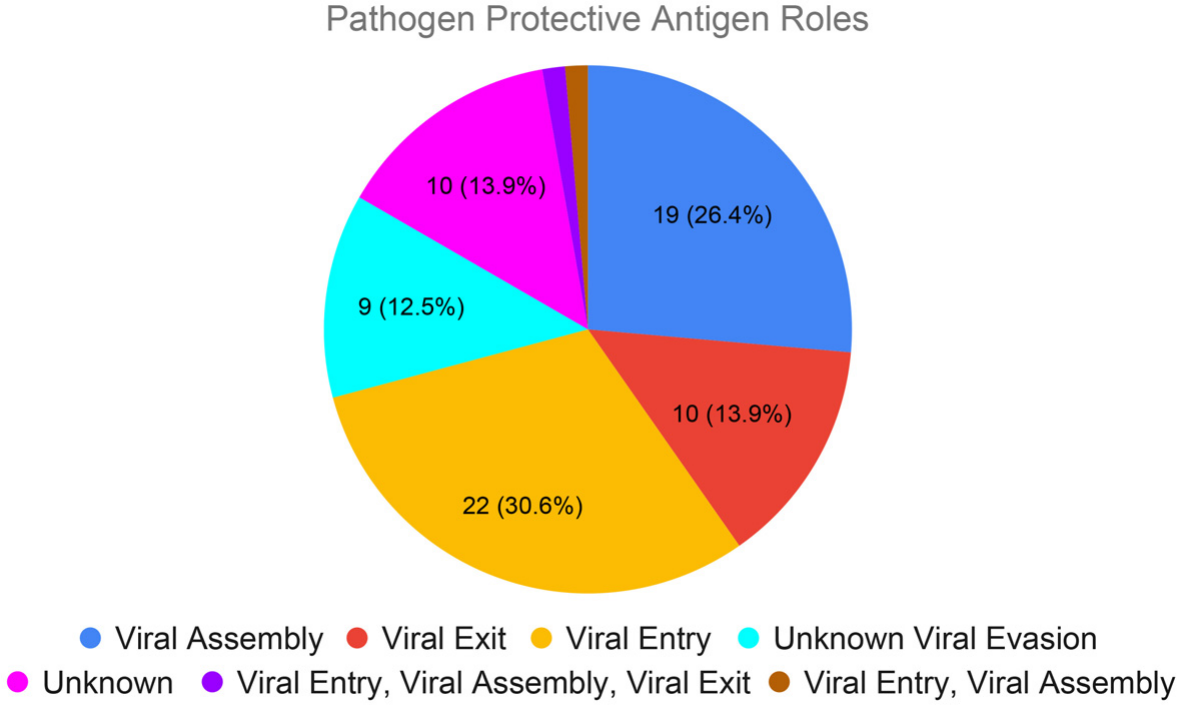
Viral life cycle stages participated by the protective antigens of top ranked human and animal viral pathogens. The 72 protective protein antigens from the top 20 human and animal viral viruses with highest numbers of vaccines collected (**Tables 1**, **2**) were analyzed here. These proteins were classified based on their roles in viral entry into a host cell, viral assembly of the capsid and replication of RNA, viral exit of the host cell, and immune evasion. Proteins without an appropriate GO annotation are listed as unknown.

To further validate this finding, we expanded this analysis to the top 10 human viral pathogens with the highest number of vaccines collected in VIOLIN (**Table 1**) and top 10 animal viral pathogens with the highest number of vaccines collected (**Table 2**). Only whole viral virus vaccines are available for canine adenovirus and canine parainfluenza virus. A total of 72 unique viral proteins serves as protective vaccine antigens for the remaining 18 viruses (**Figure 4**; **Supplementary Table S3**). Our research found that viral vaccines for these top ranked human and animal viral pathogens also target specific stages in the viral life cycles (Ryu, 2016). Out of 72 protective antigens, 22 are actively involved in viral entry, 19 in viral assembly, 10 with viral exit, 9 with viral evasion although details unknown, 1 protein involved in both viral entry and assembly, and 11 with unknown function (**Figure 4**). The one protein involved in both viral entry and assembly is a 110 kDa viral polyprotein (VP243, or VP2-VP4-VP3) of the Infectious Bursal Disease Virus (IBDV), which has been used in two IBDV vaccines (Heine and Boyle, 1993; Li et al., 2013). Encoded by the VP243 gene, VP243 can be self-cleaved by the viral protease VP4 to form viral proteins VP2 (48 kDa), VP3 (32 kDa), and VP4 (28 kDa) (Li et al., 2013). VP2 is active for viral entry while the other two proteins are part of viral assembly.

### 3.3 Viral vaccines targeting viral antigens at different virion locations

Our research found that viral vaccines often target either the virion or proteins located as part of it. The virion is the complete, infective form of a virus outside a host cell, with a core of RNA or DNA and a capsid. We found that many viral vaccines target the outer components of the virion, while a few did target non-structural proteins inside of the virion.

**Figure 5:**
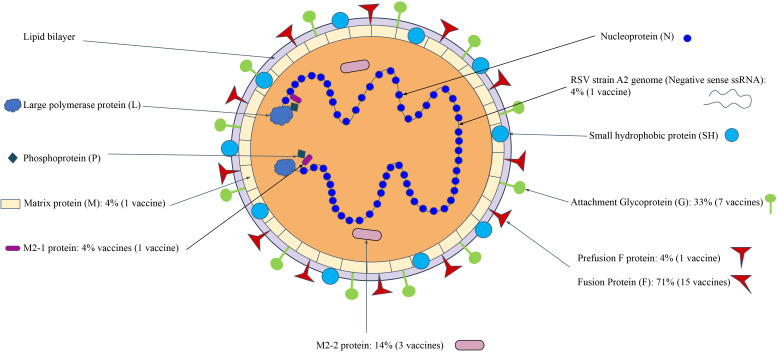
HRSV viral vaccines targeting proteins at different locations in the virion structure. The M2 and P proteins help make up the majority of the virion. The letters represent the full names of the proteins: Fusion (F) protein, Membrane (M1, M2) protein, Glycoprotein (G), Hemagglutinin-neuraminidase (HN), Nucleocapsid (N) protein, and Non–structural protein (NS). The information of figure construction is from 10.1155/2013/595768 (Bawage et al., 2013).

As an example, Human Respiratory Syncytial Viruses (HRSV) (Topalidou et al., 2023) is a member of order Mononegavirales that target the lower respiratory system, through in particular they target infantile humans (HRSV) or cattle (BRSV). These viruses have a similar structure as shown in **Figure 5**. The use of a Fusion (F protein) for viral entry has led to it being the most common protein target for HRSV vaccines (15, 71%). The other commonly used vaccine antigen is the G glycoprotein, which is located as part of the outer structure of the protein. The use of other antigens, either singly or jointly, utilize structural proteins by themselves or in conjunction with other non-structural proteins. It is also noted that multiple antigens might be used together as a cocktail vaccine (Russell and Hurwitz, 2021).

It is clear that both structural and non-structural viral proteins have been used for viral vaccine development. As such, we wanted to see if there were any general patterns that could be discerned in terms of antigen quality based on the location of a viral protein. More analyses were then conducted as described below.

### 3.4 Systematic analysis of viral proteins using Vaxign2 and Vaxign-ML

**Figure 6:**
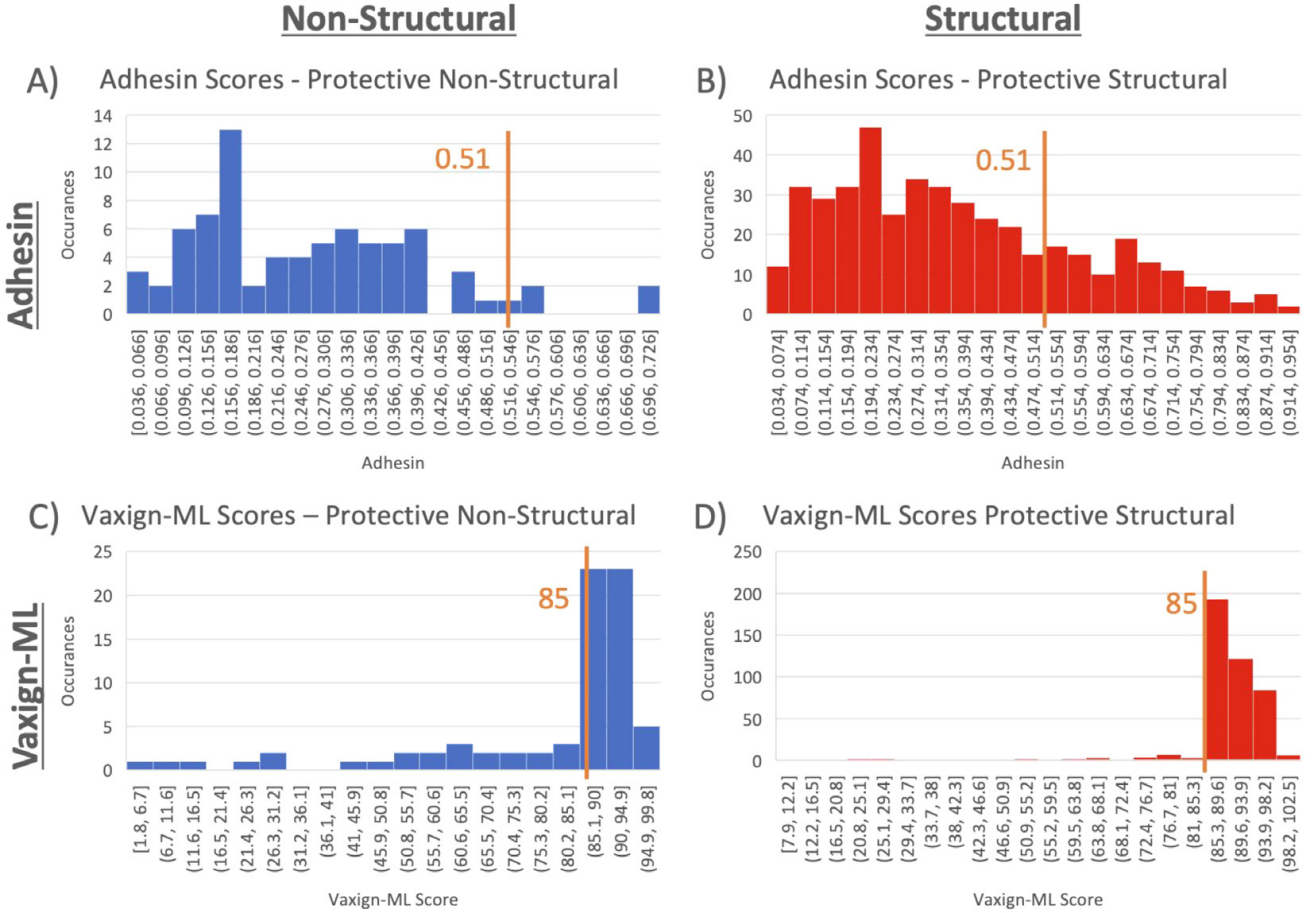
Adhesin and protegenicity score analysis of protective viral structural and nonstructural vaccine antigen proteins. Structural proteins are shown in blue, while non-structural proteins are shown in red. **(A, C)** are for adhesion measurements, and **(B, D)** are for Vaxign-ML protegenicity measurements.

Structural and non-structural protective antigens from VIOLIN were analyzed using Vaxign2 (Ong et al., 2021) to obtain Adhesin scores and using Vaxign-ML to generate protegenicity scores (**Figure 6**). Adhesin has been suggested to be a stronger indicator of protective antigen in bacterial vaccines (Ong et al., 2017a) and viral vaccines (Ong et al., 2020b). Our results found that 21.42% of protective viral vaccine antigens were predicted to be adhesins, and most of these protective viral adhesins are structural proteins (**Figures 6A, B**). Using a protegenicity score of 85% as the threshold, approximately 88% of structural proteins and 65% of non-structural proteins within the protective dataset met the criteria used to identify protective antigen candidates (**Figures 6C, D**). Non-structural proteins exhibited statistically significant lower protegenicity (p-value = 2.2E-4) and adhesin (p-value = 4.2E-6) scores in comparison to their structural counterparts.

**Figure 7:**
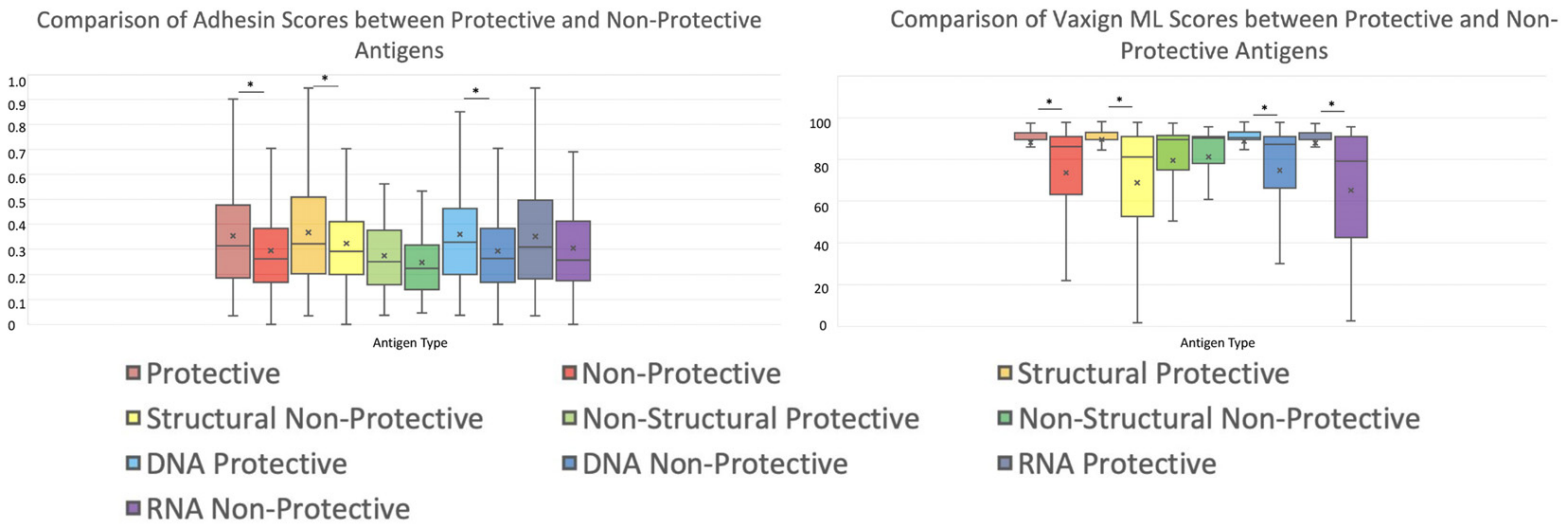
Comparison of protective antigens and non-protective antigens using Vaxign-ML. Both histograms share the legend below. Significance is calculated via Welch’s t-test. Values for this are shown as part of **Table 3**.

We further compared the adhesin scores and protegenicity scores of protective and non-protective antigens with different protein categories (**Figure 7**; **Table 3**). Our viral vaccine antigen analysis showed that predicted adhesins still have significantly higher chances of being protective antigens compared to non-adhesin proteins (**Figure 7**). Specifically, we found statistically significant differences in terms of adhesin prediction between the whole groups of protective and non-protective antigens. In the subcategories of structural and DNA virus proteins (but not in non-structural and RNA virus proteins), protective antigens had statistically significantly larger adhesin scores than non-protective antigens (**Figure 7**; **Table 3**). In terms of protegenicity scores, except for non-structural proteins, each category (including DNA virus, RNA virus, and structural proteins) that was analyzed showed that protective antigens had statistically significantly higher protegenicity scores than non-protective antigens (**Figure 7**; **Table 3**).

**Table 3:** Summarized Vaxign-ML comparisons between protective and non-protective antigens.

Category	Mean Vaxign-ML Score (Protegenicity)	Vaxign-ML T-test P-value	Mean Vaxign-ML Adhesin Score	Adhesin Welch T-test P-value
Protective Antigens	88.03	2.9E-30	0.35	3.0E-07
Nonprotective Antigens	79.49		0.29	
Protective Structural Proteins	89.52	1.4E-32	0.37	1.3E-03
Nonprotective Structural Proteins	68.81		0.28	
Protective Non-structural Proteins	79.44	5.4E-01	0.16	1.6E-1
Nonprotective Non-structural Proteins	87.19		0.14	
Protective DNA Proteins	89.60	7.8E-26	0.36	6.7E-4
Nonprotective DNA Proteins	74.66		0.26	
Protective RNA Proteins	87.79	1.7E-07	0.18	6.3E-2
Nonprotective RNA Proteins	65.06		0.17	

### 3.5 GO enrichment analysis of structural and non-structural viral vaccine antigens

Initially we performed the GO enrichment analysis using the commonly used DAVID method (Sherman et al., 2022). However, the DAVID analysis did not get any meaningful results, likely due to the sporadic distribution of viral proteins without a solid background setting from the DAVID tool. Later, we developed our own Fisher’s exact test after merging proteins based on homology. Our UniProtKB analysis identified 254 annotated structural proteins and 122 annotated non-structural proteins from the viral protein antigen list.

**Table 4** shows the most common biological process and molecular function terms from our GO enrichment analysis. Specifically, nonstructural proteins showed statistically significant greater frequency to terms related to viral assembly (DNA-binding (7.99e-31), RNA binding (p = 1.77e-16)) and cell cycles (Perturbation by virus of host G1/S transition checkpoint (3.85e-17)). Structural proteins, in contrast, had annotation functions associated with viral infection (viral entry into host cell (p = 0.01), and virion attachment to host cell (0.14e-4)). Both sets of structural and non-structural proteins did not show any significant differences in terms of the modification of host cell functions such as suppression of host type 1 interferon-mediated signaling pathway (p = 3.85e-1), and proteolysis (8.13e-1).

**Table 4:** Most common GO biological process and molecular function annotations for structural and non-structural protective proteins. Annotations were listed if they were the top 10 most common annotations for either structural proteins or non-structural proteins.

GO Annotation	# Nonstructural Proteins	# of Structural Proteins	p-value
DNA binding	69	10	7.99e-31
DNA-binding transcription factor	36	3	1.77e-16
perturbation of host G1/S transition checkpoint	31	0	3.85e-17
metal ion binding	18	0	6.43e-10
RNA binding	11	51	6.7e-1
viral entry into host cell	11	49	3.87e-1
ATP binding	10	17	7.93e-5
suppression by virus of host type 1 interferon-mediated signaling pathway	10	15	1.05-e2
modulation by virus of host apoptotic process	10	1	7.93e-5
fusion of virus membrane with host plasma membrane	3	16	1.35e-1
virion attachment to host cell	6	48	1.43e-4
fusion of virus membrane with shot endosome membrane	0	30	6.30e-6
clathrin-dependent endocytosis of virus by host cell	0	24	6.87e-5
mitigation of host antiviral defense response	1	24	7.00e-4
fusion of virus membrane with host plasma membrane	3	16	1.35e-1

The gene set enrichment for structural and nonstructural proteins P-value is given from the Fischer’s exact test used to compare both gene sets.

### 3.6 Ontological modeling and representation of viral vaccines using the vaccine ontology

**Figure 8:**
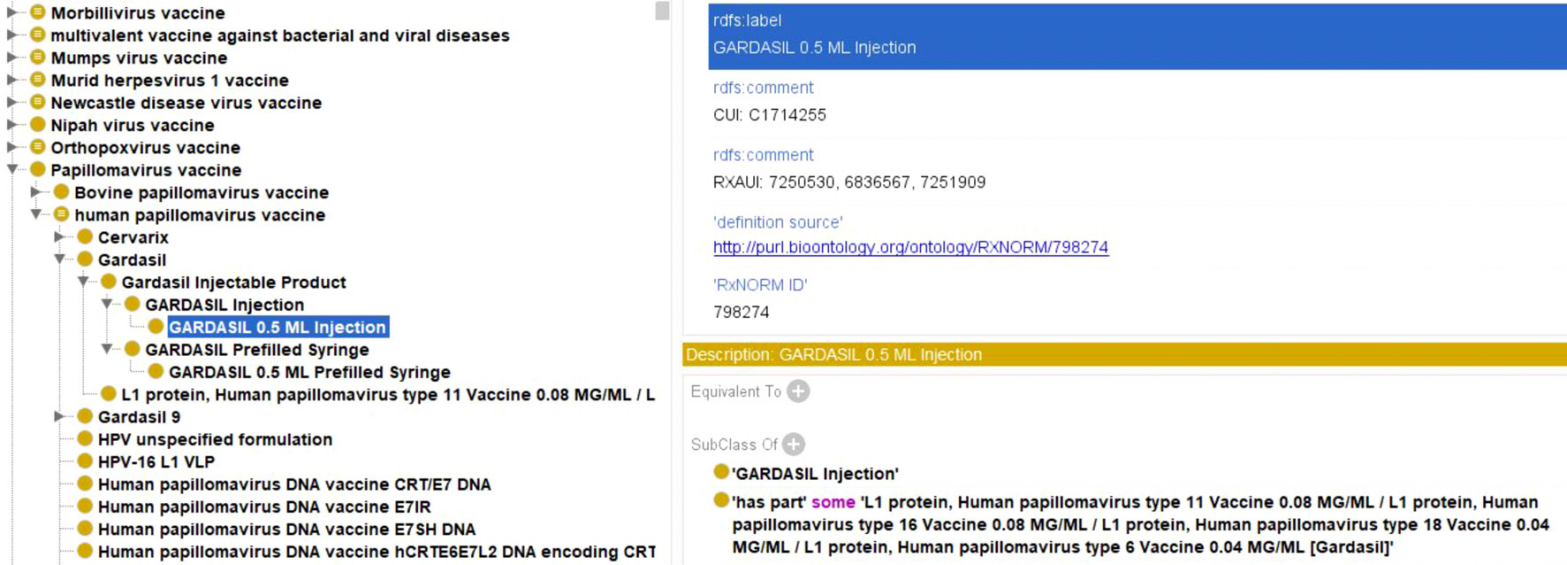
Ontological representation of Gardasil vaccine within VO. VO contains hierarchical categorization of multiple vaccines along with annotations and axioms. This formulation is also linked to the RxNORM database as part of the listed axioms.

All viral vaccines added to VIOLIN follow the template of the Vaccine Ontology (VO) to add appropriate axioms to aid representation and support structured DL queries for analysis. **Figure 8** shows the representation of Gardasil 0.5 ML injection in VO. Gardasil is a licensed multivalent HPV vaccine that targets L1 protein for HPV types 6, 11, 16, and 18 (Shi et al., 2007). Gardasil, specifically, is a cocktail of four specific L1 vaccine proteins. Therefore, the following axioms are included as part of the four related ingredient vaccines:

‘is a’ some ‘GARDASIL Injection’

‘has part’ some ‘L1 protein, Human papillomavirus type 11 Vaccine 0.08 MG/ML/L1 protein, Human papillomavirus type 16 Vaccine 0.08 MG/ML/L1 protein, Human papillomavirus type 18 Vaccine 0.04 MG/ML/L1 protein, Human papillomavirus type 6 Vaccine 0.04 MG/ML (Gardasil)’

Each of the four L1 protein related ingredient vaccines as described above is further defined in the VO.

Using a similar ontological design, VO has represented all the viral vaccines. In addition to viral vaccines, VO also includes vaccines against other pathogens such as bacteria and parasites. The OntoFox tool can be used to generate a specific subset of VO that includes only viral vaccines.

### 3.7 VIOLIN web viral vaccine query

**Figure 9:**
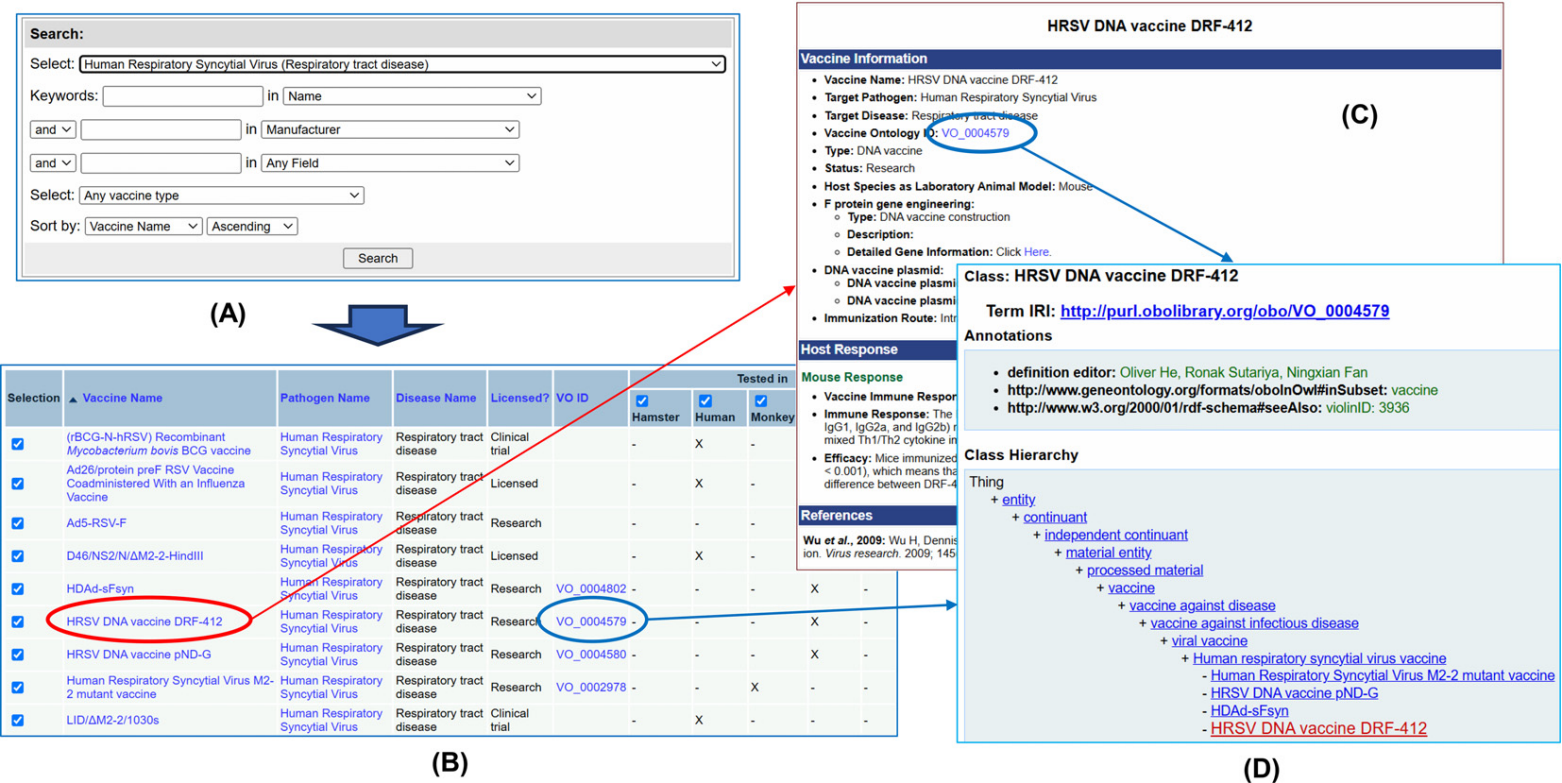
Use of Vaccine Query for HRSV DNA vaccine DRF-412. **(A)** Vaxquery can be used to select a list of vaccines based on criteria specified. **(B)** The collection of all HRSV vaccines is shown in VIOLIN displaying key information about the pathogen, disease, licensed use, and existing VO ID. **(C)** Clicking on a specific vaccine name can be done to pull more detailed information. **(D)** The use of a VO ID along linkage between VIOLIN and VO.

The VIOLIN web system provides user-friendly web query interfaces for querying and analyzing viral vaccines. **Figure 9** provides a simple demonstration on how to do so. Specifically, we can first use a simple or advanced version of the Vaxquery (i.e., a VIOLIN database query) to query and/or compare various types of viral vaccines. Vaccines can be searched by name, by species, and by vaccine platform and antigen. In this demo, after we select the HRSV in the advanced Vaxquery web interface (**Figure 9A**), Vaxquery provides the full list of HRSV vaccines (**Figure 9B**). A specific vaccine can be selected to provide more information regarding its components and experimental effects (**Figure 9C**). Alternatively, multiple vaccines can be selected for information comparison (data not shown). Each vaccine in VIOLIN is typically assigned a VO identifier. The VO identifier can also be clicked to view the detailed information (e.g., definition and axioms) about this vaccine in VO in an Ontobee (Ong et al., 2017b; He et al., 2018) web page (**Figure 9D**).

## 4 Discussion

The contributions of this article are multiple. First, we report our systematic collection and annotation of 2,847 viral vaccines against 95 viral species and their associated 542 vaccine antigens stored in our VIOLIN vaccine knowledgebase and their representation in the Vaccine Ontology. Second, we performed a systematic pattern analysis on these viral vaccines and vaccine antigens. Our pattern analysis focuses on three aspects: how the viral vaccines target viral life cycle and viral proteins in the virion structure, Gene Ontology enrichment analysis of common patterns in these viral antigens, and reverse vaccinology (Rappuoli et al., 2016; Ong and He, 2022) assessment of the roles of adhesin and protegenicity scores in protective viral antigen prediction. Lastly, we provide a web query demonstration to show how the viral vaccines can be queried and analyzed on the VIOLIN website.

To the best of our knowledge, VIOLIN remains the only database with a systematic collection of viral vaccines and antigens. There are databases focused on collections of different viruses and viral strains such as GISAID for influenza and SARS-CoV-2 viral variants (Shu and Mccauley, 2017) and the Bacterial and Viral Bioinformatics Resource Center (BV-BRC) as resources of viral genes and proteins (Olson et al., 2023). However, these resources do not focus on the topic of viral vaccines. While we have also developed the Cov19VaxKB (Huang et al., 2021; Guo et al., 2022), which provides a comprehensive collection of various COVID-19 vaccines, it is a sub-database under the VIOLIN vaccine resources. Overall, our VIOLIN viral vaccine collection thus provides a unique resource for viral vaccine annotation and analysis.

To better understand how these viral vaccines function, we have a series of pattern analysis in order to identify enriched or unique features in these viral vaccines and their associated components including the viral antigens. First, we analyzed how viral vaccines are related to different viral life cycle stages and how they target viral proteins at different virion structure locations. As illustrated in our analysis of the HPV vaccines and vaccines in those highly ranked human and animal pathogens based on the numbers of vaccines associated with these pathogens (**Tables 1**, **2**), our study found that indeed different vaccines are often developed against proteins at different life cycle stages of the viral pathogens. Meanwhile, as shown in the HRSV example, different viral vaccines target proteins at different virion locations such as the structural surface proteins and non-structural nucleocapsid proteins. Overall, our results showed that structural surface proteins are better vaccine candidates than non-structural proteins in vaccine design and development.

Our Gene Ontology (GO) enrichment analysis is novel in that it overcomes the limitation of the lack of overall background count in the typical enrichment analysis. Each viral genome is typically small. For example, the HRSV virus only has a genome of 10 proteins. SARS-CoV-2, a more complex virus, has 29 distinct proteins (Bai et al., 2022). Influenza, the most prevalent virus that we have vaccines for, has 8 distinct proteins across 5 major strains (Bouvier and Palese, 2008). In this case, we were not able to use the popular DAVID gene expression enrichment tool (Sherman et al., 2022) to perform the Gene Set Enrichment Analysis. To address this issue, we realized that multiple antigens in various viral species are homologous to each other, or they are variants of the same proteins. Of the 542 viral antigens, there are 118 duplicates of 33 proteins, the largest set being 21 hemagglutinin proteins from different influenza strains. Our GO enrichment analysis merged proteins based on homology and used a Fisher’s exact test to calculate the enrichment values for individual GO terms. Finally, our GO enrichment analysis found that top 10 most common biological process and molecular function terms associated with our collected protective structural and non-structural viral protein antigens (**Table 4**), such as perturbation by virus of host G1/S transition checkpoint, viral entry into host cell, and suppression of host type 1 interferon-mediated signaling pathway. In addition, different types of binding activities were enriched, including DNA binding, RNA binding, ATP binding, and metal ion binding (**Table 4**), demonstrating the important role of viral proteins related to these viral binding activities in the stimulation of protective immunity against viral diseases. It is possible to later consider these functional categories as features for protective viral antigen prediction.

To support more effective protective viral antigen prediction, we used the reserve vaccinology tools Vaxign2 and Vaxign-ML with the goal to identify potential patterns from the viral antigens. Our previous studies show a high value of using adhesin probability as a predictor for bacterial vaccine antigens (Ong et al., 2017a) and COVID-19 viral vaccines (Ong et al., 2020b). Therefore, given the large number of viral vaccine antigens available, it would be interesting to examine the general pattern of how adhesin probability is associated with viral vaccines. Overall, only 21.42% of protective viral vaccine antigens were predicted to be adhesins. Most of these protective viral adhesins are structural proteins, and non-structural proteins exhibited statistically significant lower adhesin scores than structural counterparts. Compared to non-protective antigens, predicted adhesins still have significantly higher chances of being protective antigens, esp. in the subcategories of structural and DNA virus proteins. Structural proteins tended to have higher adhesin probability, likely due to these proteins being responsible for the virion. However, the large amount of non-structural protective antigens suggests that additional prediction factors may prove to be more viable. The GO analysis of non-structural protective antigens suggests identification of a viral protein’s activity during a cell cycle may serve as a better predictor for non-structural protective antigens.

Our analysis confirmed the value of the Vaxign-ML “protegenicity” score (Ong et al., 2020a) to be used for viral vaccine candidate prediction. The original Vaxign-ML studies (Ong et al., 2020a, 2020b) suggested that a cutoff of 0.90 would be a good prediction threshold for the protegenicity score. However, our study showed the cutoff of 85% appears to be good for protective viral antigen prediction. Our future systematic work is needed to investigate such a cutoff for viral vaccine antigen prediction.

Vaccine design for species that lack traditional vaccines, such as HIV and Hepatitis C viruses, are trickier due to either anti-immune evasion mechanisms or high rates of mutability. HIV utilizes glycoproteins to hide epitopes used for neutralizing and antibodies. Hepatitis C, in contrast, is more similar to influenza in that there are over 67 strains that are sufficiently different that a vaccine antigen useful against one strain may not be useful against the others. Our collection has provided many promising vaccine candidates and vaccine antigen candidates. As for antigen targets, recent studies have shown that the structural envelope proteins of both HIV and Hepatitis C can be used to induce an immunogenic response (Gomez-Escobar et al., 2023; Saunders et al., 2024). To develop effective vaccines for these viruses, more research is needed to identify conserved protective antigens (including epitopes) and possibly improve the vaccine antigen delivery systems as well. To address the fast-evolving mutants of viruses, it may also be feasible to develop personalized therapeutic vaccines that are able to use specific mutant antigens from the patients for personalize vaccine development and usage. The successful use of therapeutic vaccines for cancer, which have similar evasion of the host immune system, have been recorded within VIOLIN (Asfaw et al., 2024; Zheng et al., 2024).

Our study also has limitations. First, our collection of viral vaccines and protective viral antigens is not comprehensive and does not include many new viral vaccines being reported in the literature. However, we believe that our manually collected and annotated 2,847 viral vaccines, 542 vaccine antigens, and their associated information have provided sufficient information for generating many meaningful insights. To make our work more comprehensive, we plan to collect the information of more vaccines in the future, including the usage of large language models (LLMs) for more efficient literature mining and annotation (Li et al., 2024). One other limitation is that our study was solely *in silico* analysis. However, our analysis was based on our manual collection of experimentally identified results. Our work also verified vaccine prediction tools and features and generated testable hypotheses. We believe that our work is novel and provide insights to the viral vaccine antigen prediction, protective mechanism understanding, and future vaccine development.

Our future directions include systematic collection and annotations of more viral vaccines, their associated vaccine antigens, vaccine formulations, and more specific enriched patterns. For example, we would like to investigate if there are any specific patterns in terms of immune epitopes in these viral vaccine antigens. Using these viral antigens as the positive gold standards, we would also like to develop new reverse vaccinology or machine learning techniques to rationally predict and design effective viral vaccines to support public health.

## Data Availability

The original contributions presented in the study are included in the article/**Supplementary Material**. Further inquiries can be directed to the corresponding author.

## Supplementary material

Supplementary File 1 Summary of viral vaccines by clade. A supplemental file containing the different viral orders that have vaccines, with the number of vaccines per virus identified in parenthesis.

Supplementary File 2 Viral protein analysis. A supplemental file containing information generated by Vaxign-ML for protegenicity and adhesin scores. Classification of each viral protein as structural or non-structural or RNA or DNA is included. Justification for if a protein is structural or non-structural is included.

Supplementary File 3 Classification of protective viral proteins based on their roles in the viral life cycle. Different roles were assigned to the 72 proteins from the top 20 human and animal viral viruses with the highest numbers of vaccines collected (**Tables 1**, **2**). These proteins were classified based on their roles in viral entry into a host cell, viral assembly of the capsid and replication of RNA, viral exit of the host cell, and immune evasion. Proteins without an appropriate GO annotation are listed as unknown.
